# Species Diversity, Phylogeny, Divergence Time, and Biogeography of the Genus *Sanghuangporus* (Basidiomycota)

**DOI:** 10.3389/fmicb.2019.00812

**Published:** 2019-04-17

**Authors:** Lin Zhu, Jie Song, Jun-Liang Zhou, Jing Si, Bao-Kai Cui

**Affiliations:** Institute of Microbiology, Beijing Forestry University, Beijing, China

**Keywords:** biogeography, Hymenochaetaceae, medicinal mushroom, molecular clock, phylogeny

## Abstract

“Sanghuang” is a popular fungus used as a Chinese traditional medicine. In fact, it represents a group of fungi belonging to the genus *Sanghuangporus*, but little is known about its origin and biogeography. The aim of this study was to characterize the molecular relationships, origin and biogeographical distribution of *Sanghuangporus*. The multi-locus phylogenetic analyses were used to infer the phylogenetic relationships. In addition, based on Bayesian evolutionary analysis using sequences from the internal transcribed spacer (ITS), nuclear large subunit rDNA (nLSU), translation elongation factor 1-α (EF1-α), and the largest and second largest subunits of RNA polymerase II (RPB1 and RPB2), we used a fungus fossil-based approach to gain insight into the divergence time of species in *Sanghuangporus*. The molecular phylogeny strongly supports the monophyly of *Sanghuangporus* (MP = 100%, ML = 100%, and BPP = 1.00), and 13 species are recognized in this genus. The Bayesian uncorrelated lognormal relaxed molecular clock using BEAST and reconstructed ancestral areas indicate that the maximum crown age of *Sanghuangporus* is approximately 30.85 million years. East Asia is the likely ancestral area (38%). Dispersal and differentiation to other continents then occurred during the late Middle Miocene and Pliocene. The ancestor of *Sanghuangporus* probably originated in palaeotropical Northeast Asia and covered Northeast Asia and East Africa during the Oligocene-Miocene, hosted by plants that expanded via the “Gomphotherium Landbridge.” Six kinds of dispersal routes are proposed, including intercontinental dispersal events of three clades between Northeast Asia and East Africa, between East Asia and North America, and between Northeast Asia and Europe.

## Introduction

The genus *Sanghuangporus* Sheng H. Wu, L.W. Zhou, and Y.C. Dai belongs to the order Hymenochaetales, and its species are prized as medicinal fungi. The medicinal functions of *Sanghuangporus* are antitumor, antioxidation, anti-inflammatory, hypoglycemic effect, hepatoprotective effect, and improving immunity (Dai et al., 2009; Lee et al., 2015; Zhu and Cui, 2016; Lin et al., 2017). Species in this genus form parasitic relationships with *Alnus, Juglans, Lonicera, Morus, Populus, Quercus, Syringa*, and *Weigela* (Dai, 2010; Wu et al., 2012). In the past few years, several studies have examined the phylogenetic relationships within *Sanghuangporus* (Dai, 2010; Wu et al., 2012; Vlasák et al., 2013; Tian et al., 2013; Ghobad-Nejhad, 2015; Tomsovsky, 2015; Zhou et al., 2016; Zhu et al., 2017). Thirteen species are currently accepted in the genus: *S. alpinus* (Y.C. Dai and X.M. Tian) L.W. Zhou and Y.C. Dai, *S. baumii* (Pilát) L.W. Zhou and Y.C. Dai, *S. ligneus* Ghobad-Nejhad, *S. lonicericola* (Parmasto) L.W. Zhou and Y.C. Dai, *S. lonicerinus* (Bondartsev) Sheng H. Wu, L.W. Zhou and Y.C. Dai, *S. microcystideus* (Har. and Pat.) L.W. Zhou and Y.C. Dai, *S. pilatii* (Cerný) Tomšovský, *S. quercicola* L. Zhu and B.K. Cui, *S. sanghuang* (Sheng H. Wu, T. Hatt., and Y.C. Dai) Sheng H. Wu, L.W. Zhou, and Y.C. Dai, *S. vaninii* (Ljub.) L.W. Zhou and Y.C. Dai, *S. weigelae* (T. Hatt. and Sheng H. Wu) Sheng H. Wu, L.W. Zhou and Y.C. Dai, *S. weirianus* (Bres.) L.W. Zhou and Y.C. Dai and *S. zonatus* (Y.C. Dai and X.M. Tian) L.W. Zhou and Y.C. Dai (Dai, 2010; Wu et al., 2012; Tian et al., 2013; Vlasák et al., 2013; Ghobad-Nejhad, 2015; Tomsovsky, 2015; Zhou et al., 2016; Zhu et al., 2017).

Previous phylogenetic analyses of *Sanghuangporus* used the sequences of the internal transcribed spacer (ITS) (Wu et al., 2012; Tian et al., 2013; Vlasák et al., 2013; Ghobad-Nejhad, 2015; Tomsovsky, 2015), and these studies were mainly focused on the descriptions of new species. Recently, Zhou et al. (2016) used the ITS and the nuclear large subunit rDNA (nrLSU) to analyze *Sanghuangporus* and *Tropicoporus* L.W. Zhou and Y.C. Dai. However, a comprehensive multilocus phylogenetic analysis of the taxa in *Sanghuangporus* has not been performed.

*Sanghuangporus* exhibits a wide intercontinental disjunct distribution, and the majority of species are in the Northern Hemisphere (Zhou et al., 2016; Zhu and Cui, 2016). Molecular biogeography studies have provided important insights into the histories of “species” range changes (Eastwood et al., 2011; Feng et al., 2012), and several origin and biogeography studies of fungi have focused on biogeographic distribution and geological events (Floudas et al., 2012; Cai et al., 2014; Chen et al., 2015; Song and Cui, 2017). However, there has been no the estimation of divergence time and no examination of the biogeography of the *Sanghuangporus* mushrooms.

In this study, the sequences of the ITS, nrLSU, translation elongation factor 1-α (EF1-α), and the largest and second largest subunits of RNA polymerase II (RPB1 and RPB2) were used to study the phylogeny of *Sanghuangporus*. Furthermore, the divergence time between Ascomycota and Basidiomycota (582 Mya), as estimated based on fossil evidence of *Paleopyrenomycites devonicus* by Taylor et al. (2004), was used as a calibration point to estimate divergence time and explore the biogeography of *Sanghuangporus*.

## Materials and Methods

### Taxa Sampling

Sixty two samples of *Sanghuangporus* species from Europe, North America, East Asia, Central Asia, West Asia, and East Africa were studied for their morphological characters. The specimens and cultures were obtained from the herbaria of the Institute of Microbiology, Beijing Forestry University (BJFC) and the Institute of Applied Ecology, Chinese Academy of Sciences (IFP). The detailed information and GenBank accession numbers of the samples are given in Table 1.

**Table 1:** Information of sequences used in this study.

Species	Sample no.	GenBank accessions				
		ITS	nrLSU	EF1-α	RPB1	RPB2
*Auricularia* sp.	PBM 2295	DQ200918	AY634277	DQ408144	–	DQ366278
*Boletopsis leucomelaena*	PBM 2678	DQ484064	DQ435797	GU187763	GU187494	GU187820
*Calocera cornea*	AFTOL-ID 438	AY789083	AY701526	AY881019	AY857980	AY536286
*Coltricia perennis*	AFTOL-ID 447	DQ234561	AF287854	AY885147	AY864867	AY218526
*Cryptococcus humicola*	AFTOL-ID 1552	DQ645516	DQ645514	DQ645519	DQ645518	DQ645517
*Dacryopinax spathularia*	AFTOL-ID 454	AY854070	AY701525	AY881020	AY857981	AY786054
*Echinodontium tinctorium*	AFTOL-ID 455	AY854088	AF393056	AY885157	AY864882	AY218482
*Fomitiporella chinensis*	Cui 11230	KX181309	MF772796^a^	MF977774^a^	MF972230^a^	–
*Fomitiporia mediterranea*	AFTOL-ID 688	AY854080	AY684157	AY885149	AY864869	AY803748
*Inonotus griseus*	Dai 13436	KX364802	KX364823	MF977775^a^	KX364871	KX364919
*I. henanensis*	Dai 13157	KP030783	KX832918	MF977776^a^	MF972231^a^	MF973468^a^
*Lactarius deceptivus*	AFTOL-ID 682	AY854089	AY631899	AY885158	AY864883	AY803749
*Marasmius aurantiidisca*	AFTOL-ID 1685	DQ490646	DQ470811	GU187727	DQ447927	DQ474122
*M. rotula*	AFTOL-ID 1505	DQ182506	DQ457686	GU187723	DQ447922	DQ474118
*Neofavolus mikawai*	Cui 11152	KU189773	KU189804	KU189919	KU189888	KU189986
*Phylloporia pendula*	Cui 13876	MF410320	KX901670	MF977777^a^	MF972234^a^	MF973471^a^
*P. pseudopectinata*	Cui 13746	MF410322	KX242355	MF977778^a^	MF972235^a^	MF973472^a^
*Polyporus squamosus*	AFTOL-ID 704	DQ267123	AY629320	DQ028601	DQ028601	DQ408120
*Porodaedalea himalayensis*	Cui 9620	KX673605	MF772797^a^	KX852286	MF972232^a^	MF973469^a^
*P. himalayensis*	Cui 9618	KX673604	MF772798^a^	KX852285	MF972233^a^	MF973470^a^
*Rhizopus stolonifer*	CBS 609.82	AB113023	DQ273817	AB512268	AFTOL database	AFTOL database
*Sanghuangporus alpinus*	Cui 12485	MF772781^a^	MF772799^a^	MF977779^a^	MF972236^a^	MF973473^a^
*S. alpinus*	Cui 12444	MF772782^a^	MF772800^a^	MF977780^a^	MF972237^a^	MF973474^a^
*S. alpinus*	Cui 12474	MF772783^a^	MF772801^a^	MF977781^a^	MF972238^a^	MF973475^a^
*S. baumii*	Dai 16900	MF772785^a^	MF772802^a^	MF977782^a^	MF972239^a^	MF973476^a^
*S. baumii*	Cui 11769	MF772784^a^	MF772803^a^	MF977783^a^	MF972240^a^	MF973477^a^
*S. baumii*	SFC 960405-4	AF534068	–	–	–	–
*S. lignerus*	MG 12	KR073081	–	–	–	–
*S. lignerus*	MG 13	KR073082	–	–	–	–
*S. lonicericola*	Cui 10994	MF772786^a^	MF772804^a^	MF977784^a^	MF972241^a^	MF973478^a^
*S. lonicericola*	Dai 8376	KP030772	MF772805^a^	MF977785^a^	MF972242^a^	MF973479^a^
*S. lonicericola*	TAA 105317	JN642572	–	–	–	–
*S. lonicerinus*	Dai 17095	MF772787^a^	MF772806^a^	MF977786^a^	MF972243^a^	MF973480^a^
*S. lonicerinus*	Dai 17093	MF772788^a^	MF772807^a^	MF977787^a^	MF972244^a^	MF973481^a^
*S. lonicerinus*	TAA 55428	JN642575	–	–	–	–
*S. microcystideus*	O 915609	KP030787	–	–	–	–
*S. microcystideus*	AM 19	JF895465	JQ910907	–	–	–
*S. microcystideus*	AM 08	JF895464	JQ910906	–	–	–
*S. pilatii*	BRNM 771989	KT428764	KT428765	–	–	–
*S. quercicola*	Li 1149	KY328312	MF772808^a^	MF977788^a^	–	–
*S. quercicola*	Dai 13947	KY 328309	MF772809^a^	MF977789^a^	MF972245^a^	MF973482^a^
*S. sanghuang*	Cui 14419	MF772789^a^	MF772810^a^	MF977790^a^	MF972246^a^	MF973483^a^
*S. sanghuang*	Cui 14420	MF772790^a^	MF772811^a^	MF977791^a^	MF972247^a^	MF973484^a^
*S. vaninii*	Dai 8236	MF772791^a^	MF772812^a^	MF977792^a^	MF972248^a^	MF973485^a^
*S. vaninii*	Cui 9939	MF772792^a^	MF772813^a^	MF977793^a^	MF972249^a^	MF973486^a^
*S. vaninii*	Cui 14082	MF772793^a^	MF772814^a^	MF977794^a^	MF972250^a^	MF973487^a^
*S. vaninii*	DMR 95-1-T	KU139198	KU139258	KU139380	–	KU139318
*S. weigelae*	Dai 16077	MF772794^a^	MF772815^a^	MF977795^a^	MF972251^a^	MF973488^a^
*S. weigelae*	Dai 15770	MF772795^a^	MF772816^a^	MF977796^a^	MF972252^a^	MF973489^a^
*S. weirianus*	IMSNU 32021	AF110989	–	–	–	–
*S. weirianus*	CB 618.89	AY558654	AY059035	–	–	–
*S. zonatus*	Dai 10841	JQ860306	KP030775	MF977797^a^	MF972253^a^	MF973490^a^
*S. zonatus*	Cui 8327	JX069837	MF772817^a^	MF977798^a^	–	MF973491^a^
*Schizosaccharomyces pombe*	972h-	Z19578	Z19136	NM001021161	NM001021568	NM001018498
*Serpula lacrymans*	REG 383	GU187542	GU187596	GU187752	GU187485	GU187809
*Suillus pictus*	AFTOL-ID 717	AY854069	AY684154	AY883429	AY858965	AY786066
*Tropicoporus dependens*	JV 0409/12-J	KC778777	MF772818^a^	MF977799^a^	MF972254^a^	MF973492^a^
*T. guanacastensis*	O 19228	KP030794	MF772819^a^	MF977780^a^	–	–
*T. linteus*	JV 0904/64	JQ860322	JX467701	MF977781^a^	–	–
*T. pseudolinteus*	JV 0402/35-K	KC778781	MF772820^a^	MF977782^a^	–	–
*T. sideroxylicola*	JV 0402/30-J	KC778782	–	–	–	–
*Ustilago maydis*		AY854090	AF453938	AY885160	AFTOL database	AY485636

*^*a*^Newly generated sequences.*

Macro-morphological descriptions were based on the field notes. Special color terms followed Petersen (1996). Micro-morphological data were obtained from the dried specimens, and observed under a light microscope following Han et al. (2016). Sections were studied at a magnification of up to ×1000 using a Nikon Eclipse 80i microscope and phase contrast illumination. Spores were measured from sections cut from the tubes. In presenting the variation of spore size, 5% of measurements were excluded from each end of the range, and were given in parentheses. The following abbreviations were used: KOH = 5% potassium hydroxide, CB = Cotton Blue, CB+ = cyanophilous, CB- = acyanophilous, IKI = Melzer’s reagent, IKI- = neither amyloid nor dextrinoid, L = mean spore length (arithmetic average of all spores).

### DNA Extraction, PCR, and DNA Sequencing

Genomic DNA was extracted from dried specimens and cultures using CTAB rapid plant genome extraction kit (Demeter Biotech Co., Ltd, Beijing) according to the manufacturer’s instructions with modifications. Five DNA gene fragments were analyzed, including those coding for RPB1, RPB2 and EF1-α, along with two non-protein coding regions: ITS and nrLSU. The primer pairs ITS5/4, LR0R /LR7, 983F/1567R, Af/Cf, and 5F/7CR listed in Table 2 were used to amplify ITS, nrLSU, EF1-α, RPB1, and RPB2, respectively (Vilgalys and Hester, 1990; Liu et al., 1999; Matheny et al., 2002; Rehner and Buckley, 2005; Binder et al., 2010). To improve the success rate of RPB2 amplification, a new primer pair, R2s-1 (CCTCGTTACGGGCTTGTT) and R2a-1 (AGCATTTGGAAGTGCCTTG), was designed based on eleven obtained sequences using Primer-Premier 5 (Premier Biosoft International, Palo Alto, CA, United States). PCR was performed in a reaction mixture containing 25 μl of 2 × EasyTaq^®^ PCR SuperMix, 2 μl of Forward Primer (10 μM), 2 μl of Reverse Primer (10 μM), and 2 μl of Template DNA. The total volume was adjusted to 50 μl with sterile deionized H_2_O. The PCR amplifications were conducted using an Eppendorf Master Cycler (Eppendorf, Netheler-Hinz, Hamburg, Germany). The PCR procedure for ITS was: initial denaturation at 95°C for 2 min, followed by 35 cycles of denaturation at 94°C for 45 s, annealing at 53°C for 45 s and extension at 72°C for 2 min, and a final extension at 72°C for 10 min. The PCR procedure for nrLSU was: initial denaturation at 94°C for 5 min, followed by 35 cycles of denaturation at 94°C for 1 min, annealing at 50°C for 1 min and extension at 72°C for 90 min, and a final extension at 72°C for 10 min. The PCR procedure for EF1-α was: initial denaturation at 95°C for 2 min, followed by 35 cycles of denaturation at 94°C for 45 s, annealing at 56°C for 45 s and extension at 72°C for 2 min, and a final extension at 72°C for 10 min. The PCR procedure for RPB1 and RPB2 was: initial denaturation at 94°C for 2 min, followed by 36 cycles of denaturation at 94°C for 45 s, annealing at 53°C for 90 s and extension at 72°C for 90 s, and a final extension at 72°C for 10 min. The PCR products were visualized by agarose gel electrophoresis and stored at -20°C after visualization. The PCR products were purified and sequenced at the Beijing Genomics Institute (China) using the same primers. 116 sequences of *Sanghuangporus* used in this paper, and 90 sequences of *Sanghuangporus* were newly generated, including 14 ITS (44% new), 19 nrLSU (76% new), 20 EF1-α (95% new), 18 RPB1 (100% new), and 19 RPB2 (95% new). All newly generated sequences were deposited in GenBank^1^.

**Table 2:** PCR primers used in this study.

Gene^∗^	Primer	Primer sequences (5′–3′)^a^
ITS	ITS5	GGA AGT AAA AGT CGT AAC AAG G
	ITS4	TCC TCC GCT TAT TGA TAT GC
nrLSU	LR0R	ACC CGC TGA ACT TAA GC
	LR7	TAC TAC CAC CAA GAT CT
EF1-α	EF1-983F	GCY CCY GGH CAY CGT GAY TTY AT
	EF1-1567R	ACH GTR CCR ATA CCA CCR ATC TT
RPB1	RPB1-Af	GAR TGY CCD GGD CAY TTY GG
	RPB1-Cf	CCN GCD ATN TCR TTR TCC ATR TA
RPB2	RPB2-5F	GAY GAY MGW GAT CAY TTY GG
	RPB2-7CR	CCC ATR GCT TGY TTR CCC AT

*^*a*^Degeneracr codes: W = A or T, R = A or G, Y = C or T, N = A or T or C or G, D = G or A or T, M = A or C. ^∗^ITS, internal transcribed spacer region; nrLSU, the large nuclear ribosomal RNA subunit; RPB1, the largest subunit of RNA polymerase II; RPB2, the second subunit of RNA polymerase II.*

### Sequence Alignments and Phylogenetic Analyses

Phylogenetic analyses were applied to the dataset that contained the ITS+nrLSU+EF1-α+RPB1+RPB2 sequences. Settings for phylogenetic analyses followed Shen et al. (2019). The five genes were initially aligned separately using Clustal Omega (Sievers et al., 2011) and then manually optimized in BioEdit (Hall, 1999). The missing sequences were coded as “N.” Ambiguous nucleotides were coded as “N.” Ambiguous sequences at the start and the end were deleted and gaps were adjusted to optimize the alignment. Ambiguously aligned regions were excluded from subsequent analyses. Finally, the five gene fragments were concatenated with SEAVIEW 4. One thousand partition homogeneity test (PHT) replicates of the ITS, nrLSU, EF1-α, RPB1, and RPB2 sequences were tested by PAUP^∗^ version 4.0 beta 10 (Swofford, 2002) to determine whether the partitions were homogeneous. The PHT results indicated all the DNA sequences display a congruent phylogenetic signal (*P*-value = 1). The sequences of *Inonotus griseus* L.W. Zhou and *I. henanensis* Juan Li and Y.C. Dai were used as outgroups (Zhou and Wang, 2015). Sequence alignments were deposited at TreeBase (submission ID 21569^2^).

Maximum parsimony (MP) analysis was performed in PAUP^∗^ version 4.0 beta 10 (Swofford, 2002). All characters were equally weighted, and gaps were treated as missing data. Trees were inferred using the heuristic search option with TBR branch swapping and 1000 random sequence additions. Max-trees was set to 5000, branches of zero length were collapsed, and all parsimonious trees were saved. Descriptive tree statistics tree length (TL), consistency index (CI), retention index (RI), rescaled consistency index (RC), and homoplasy index (HI) were calculated for each maximum parsimonious tree (MPT) generated.

For the maximum likelihood (ML) and Bayesian phylogenetic inference (BI) analyses, the optimal substitution models for the combined dataset were determined using the Akaike Information Criterion (AIC) implemented in MrModeltest 2.2 (Nylander, 2004) after scoring 24 models of evolution by PAUP^∗^ version 4.0 beta 10 (Swofford, 2002). The selected substitution models for both the combined dataset were general time reversible+proportion invariant+gamma (GTR+I+G).

The ML analysis was conducted on RAxmlGUI 1.31 (Michalak, 2012), the concatenated dataset was partitioned into five parts by sequence region, and 1000 ML searches under the GTR+GAMMA model with all model parameters estimated using the RAxmlGUI 1.31 program. The best fit maximum likelihood tree from all searches was kept. In addition, 1000 rapid bootstrap replicates were run with the GTR+CAT model to assess the reliability of the nodes.

The BI analysis was performed with MrBayes 3.2 (Ronquist and Huelsenbeck, 2003). Four Markov chains were run for 20 million generations and trees were sampled every 1000 generations. The first 25% of the sampled trees were discarded as burn-in, and the remaining ones were used to reconstruct a majority rule consensus and calculate Bayesian posterior probabilities (BPP) of the clades. Branches that received bootstrap support values for MP and ML greater than or equal to 60% and BPP greater than or equal to 0.95 were considered as significantly supported.

### Divergence Dating Analysis

Given that fossil records of fungi had been used to calibration point to estimate the divergence time for any fungal groups (Song and Cui, 2017). We used internal calibration point by placing *P. devonicus* in the subphylum Pezizomycotina, 582 Mya, divergence time between Ascomycota and Basidiomycota in the Figure 3. The estimated divergence time was constrained by the following value: the estimated divergence time between Ascomycota and Basidiomycota is at least 400 Mya (the divergence time of *P. devonicus*) (Taylor et al., 2004). A normal distribution was applied by setting the mean and the standard deviation to 582.5 and 50.15, respectively. The parameter settings for the calibrations was the same as those used in several studies (Chen et al., 2015; Song and Cui, 2017). We retrieved the sequences of four additional species – *Marasmius rotula* (Scop.) Fr., *Mycena amabilissima* Peck, *Fomitiporia mediterranea* M. Fisch., and *Coltricia perennis* (L.) Murrill – as representative taxa of the initial diversification of mushroom-forming fungi (based on the 90-million-year-old fossil, *Archaeomarasmius leggetti* Hibbett, D. Grimaldi and Donoghue) (Hibbett et al., 1997), and the divergence of the Hymenochaetaceae (based on the 125-million-year-old fossil, *Q. cranhamii* S.Y. Sm., Currah, and Stockey) (Smith et al., 2004).

The origin time of *Sanghuangporus* was estimated in BEAST v1.8.0 (Drummond and Rambaut, 2007) with the molecular clock and substitution models unlinked but with the trees linked for each gene partition. Two nuclear ribosomal RNA genes (ITS and nrLSU) and three protein coding genes (EF1-α, RPB1, and RPB2), were concatenated for molecular dating. ITS1, ITS2, and the introns in EF1-α, RPB1, and RPB2 were excluded for a conservation analysis. MrModeltest v2.2 was used to select the best models of evolution using the hierarchical likelihood ratio test (Nylander, 2004). The GTR+I+G model was used for the EF1-α+RPB1+RPB2 and the HKY+I+G model for the ITS+nrLSU data, based on the results from the Modeltest. The uncorrelated lognormal relaxed molecular clock and the Yule speciation prior set were set were used to estimate the divergence time and the corresponding credibility intervals. We approximated the posterior distributions of parameters using MCMC analysis for 50 million generations with a burn-in percentage of 10%. The BEAST input files were constructed using BEAUti (within BEAST). The convergence of the chains was confirmed using Tracer v1.6^3^, and samples from the posterior distributions were summarized on a maximum clade credibility tree with the maximum sum of posterior probabilities listed on its internal nodes using the program TreeAnnotator v1.8.0 (Drummond and Rambaut, 2007) with the posterior probability limits set to 0.8 to summarize the mean node heights. FigTree v1.4.2 (Rambaut, 2012) was used to visualize the resulting tree and to obtain the means and 95% HPD (Drummond and Rambaut, 2007). A 95% HPD marks the shortest interval that contains 95% of the values sampled.

We also estimated the divergence time of the main nodes in *Sanghuangporus* using the ITS dataset containing representatives of all 13 species. The estimated crown age of this genus inferred by the combined ITS+nrLSU and EF1-α+RPB1+RPB2 data was used as the calibration point to date the ITS phylogeny by setting the prior to a normal distribution. The other procedures were the same as the ones applied in the estimation using the combined dataset.

### Biogeographic Analysis

Reconstruction makes it possible to infer the original location and dispersal routes of the organisms (Song and Cui, 2017). The geographic distributions of ancestor lineages were defined based on plate-tectonic and dispersal paths histories (Esseghir et al., 2000; Meulenkamp and Sissingh, 2003). To infer ancestral areas, we performed Bayesian binary Markov chain Monte Carlo (BBM) analysis implemented in RASP 3.2 (Yu et al., 2015) by setting the generations to 10 million and by discarding the first 10% of samples as burn-ins; the other parameters used were the default settings. The geographic distributions for the *Sanghuangporus* were delimited into seven areas: (A) Northeast Asia, including Northeast China, Russian Far-East, Korea, and Japan, (B) South and Central China, located to south of the Qinling Mountains, (C) Central Asia, (D) West Asia, (E) North America, (F) Europe, and (G) East Africa. ArcGIS v10.1 (ArcGIS Platform^4^) was used to visualize the geographic distribution and possible dispersal routes of *Sanghuangporus*.

## Results

### Species Diversity

**FIGURE 1:**
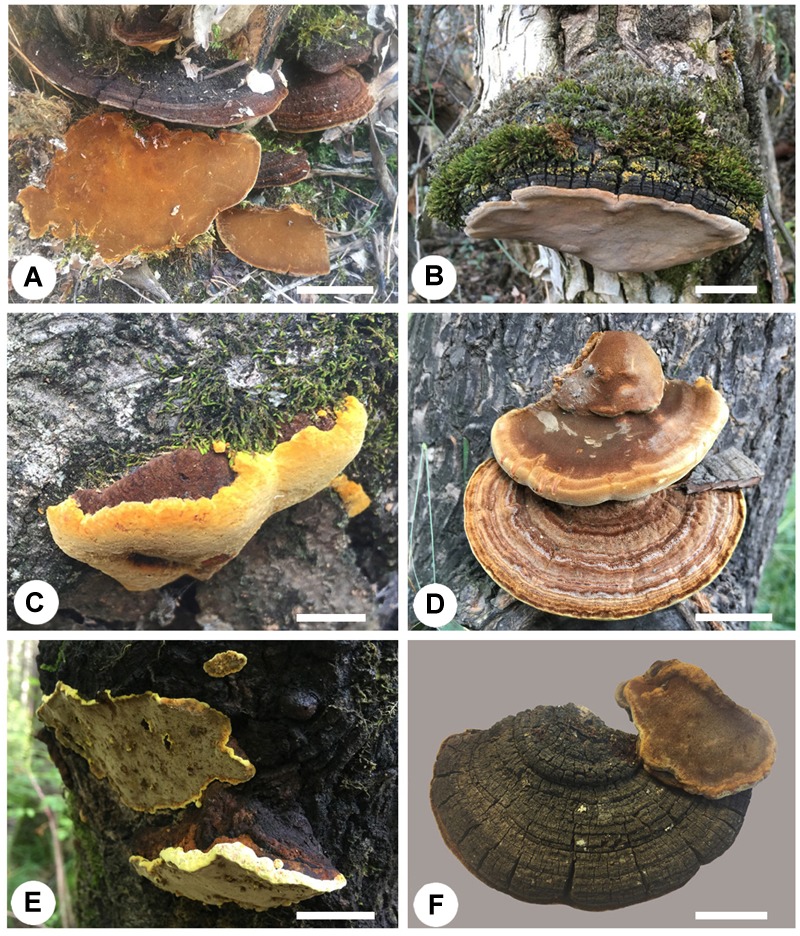
Basidiomata of *Sanghuangporus* species. **(A)** *S. alpinus*. **(B)** *S. lonicerinus*. **(C)** *S. sanghuang*. **(D)** *S. baumii*. **(E)** *S. vaninii*. **(F)** *S. lonicericola*. Bars = 1 cm.

Thirteen species of *Sanghuangporus* have been accepted. They are widely distributed across temperate to subtropical and tropical regions. Basidiomata of several *Sanghuangporus* species are shown in Figure 1. The main morphological characters and distribution details of *Sanghuangporus* are listed in Table 3.

**Table 3:** Main characteristics and distribution information of *Sanghuangporus*.

Species	Distribution	Climate	Host	Pores/	Basidiospores
				mm	(μm)
*S. alpinus*	Southwest China	Plateau climate zone	*Lonicera* and the other broadleaf trees	5–7	3.1–3.9 × 2.6–3.2
*S. baumii*	Northeast Asia	Temperate zone	*Syringa* and the other broadleaf trees	7–8	3.3–4.5 × 2.4–3.5
*S. lonicericola*	Northeast China, and Russian Far-East	Temperate zone	*Lonicera*	8–10	3.3–4.1 × 2.4–3.3
*S. zonatus*	South China	Tropic zone	Angiosperm	7–8	3.5–4.0 × 2.9–3.1
*S. sanghuang*	Northeast Asia	Temperate and subtropical zone	*Morus*	6–8	4.0–4.9 × 3.1–3.9
*S. quercicola*	Central China	Temperate zone	*Quercus*	7–9	3.0–3.9 × 2.4–2.8
*S. weigelae*	Northeast Asia	Temperate and subtropical zone	*Weigela* and the other broadleaf trees	5–7	3.0–3.8 × 2.3–3.0
*S. vaninii*	North China, and North America	Temperate zone	*Populus*	6–8	3.8–4.5 × 2.8–3.7
*S. weirianus*	North America	Subtropical zone	*Juglans*	5–7	4.0–5.5 × 3.5–4.5
*S. lonicerinus*	Central Asia	Temperate zone	*Lonicera*	4–5	3.5–4.5 × 3.0–3.5
*S. ligneous*	West Asia	Plateau climate zone	*Lonicera*	4–5	3.0–4.1 × 2.5–3.5
*S. pilatii*	Europe	Temperate zone	*Populus*	3–6	4.0–4.8 × 3.1–3.8
*S. microcystideus*	East Africa	Tropic zone	*Olea*	5–7	5.1–6.0 × 4.4–5.0

### Phylogenetic Analysis of the Combined Dataset

The combined dataset (ITS+nrLSU+EF1-α+RPB1+RPB2) has an aligned length of 3867 characters, of which 2520 are constant, 372 are variable and parsimony-uninformative, and 975 are parsimony-informative characters. In the MP analysis, 18,609,864 rearrangements were attempted, and two equal maximally parsimonious trees were retained (TL = 2268, CI = 0.770, RI = 0.806, RC = 0.621, HI = 0.230). The best model for this alignment set used for estimation and applied in the Bayesian phylogenetic inference (BI) was general time reversible+proportion invariant+gamma (GTR+I+G). The Bayesian analysis resulted in a similar topology, with an average standard deviation of split frequencies = 0.002972.

**FIGURE 2:**
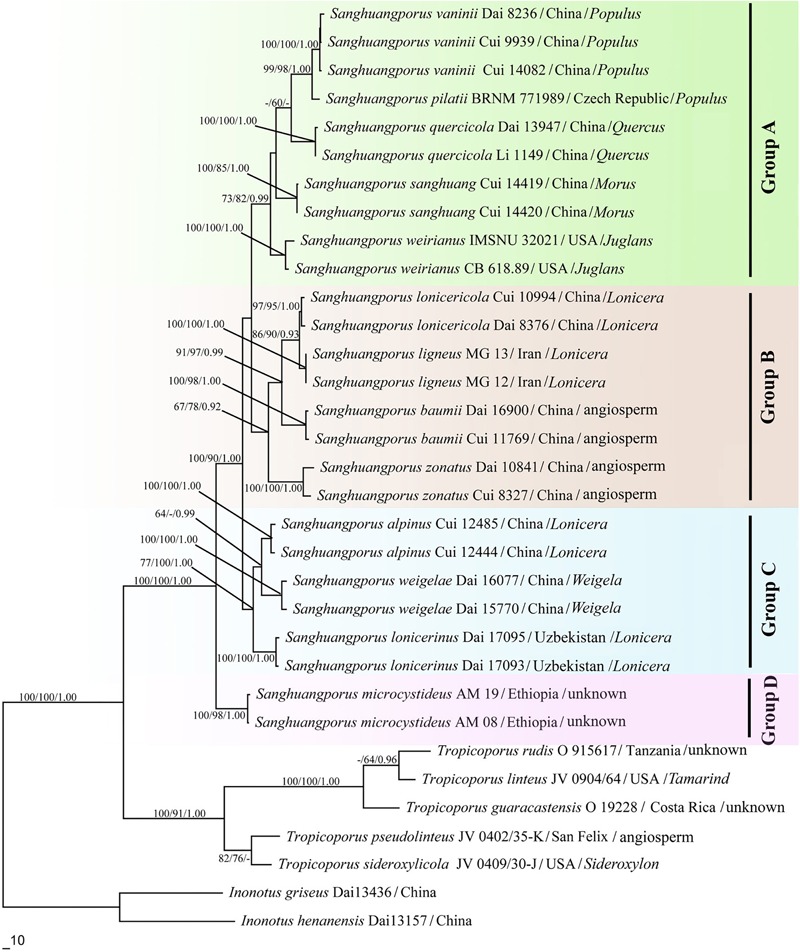
Phylogenetic consensus tree inferred from the maximum likelihood (ML) analysis based on a concatenated, multi-locus dataset (ITS+nrLSU+EF1-α+RPB1+RPB2). Branches are labeled where MP/ML support is greater than 60% and collapsed below that support threshold. BPP is labeled where greater than 0.95.

The trees obtained from the maximum likelihood (ML), MP, and Bayesian posterior probability (BPP) analyses are shown in Figure 2.

### Bayesian Estimation of Divergence Time and the Historical Biogeography of *Sanghuangporus*

**FIGURE 3:**
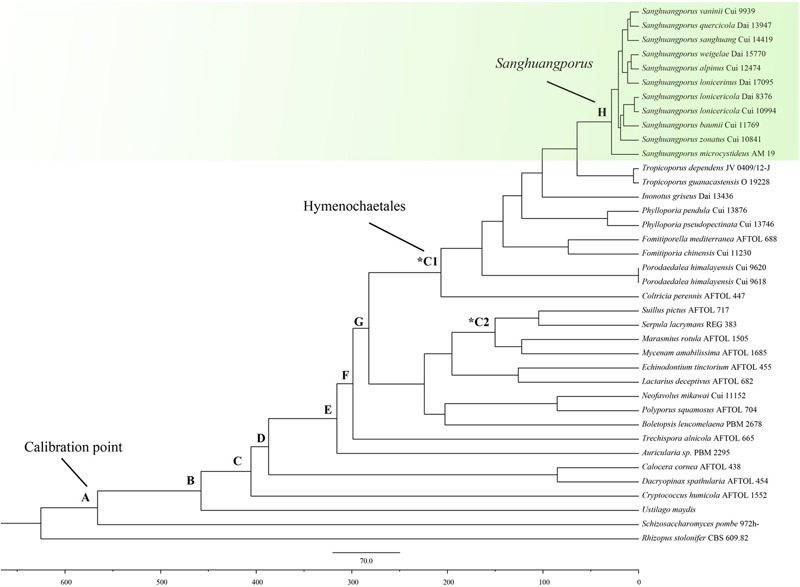
Chronogram and estimated divergence times of *Sanghuangporus* generated by molecular clock analysis using the two concatenated datasets (ITS+nrLSU and EF1-α+RPB1+RPB2) dataset. The chronogram was obtained using the Ascomycota–Basidiomycota divergence time of 582 Mya as the calibration point. The calibration point and objects of this study are marked in the chronogram. The geological time scale is in millions of years ago (Mya).

The BEAST-derived chronogram of *Sanghuangporus* (Figure 3) was based on the alignment of the two concatenated datasets (ITS+nrLSU and EF1-α+RPB1+RPB2). There were 36 taxa recovered, which were 814 and 1267 bp in length, respectively, for the two datasets. The aligned ITS dataset was 598 bp in length and was used to estimate the divergence time and biogeographical history of *Sanghuangporus*. Using the divergence time between Ascomycota and Basidiomycota (582 Mya) as a calibration point, we estimated the divergence time of Hymenochaetales as 207.21 ± 0.56 Mya (146.74–265.46 Mya, 95% higher posterior density (HPD), which was more recent than the estimate by Feng et al. (2012) (divergence time at 220.81 Mya). The divergence time of Agaricales at 123.57 ± 0.45 Mya (80.52–170.72, 95% HPD) is consistent with a previous estimate (Eastwood et al., 2011). The initial diversification of *Sanghuangporus* occurred during the Late Oligocene, 30.85 ± 0.19 Mya (15.65–37.18 Mya, 95% HPD). The divergences of the major clades within *Sanghuangporus* occurred mainly during the Late Miocene, and the divergence times of the main nodes are shown in Figure 3 and summarized in Table 4.

**Table 4:** Estimated divergence times of the main nodes.

Node	Mean ± standard	95%HPD
	error	
A: Ascomycota/Basidiomycota	564.85 ± 0.58	467.24–666.82
B: Ustilaginomycotina/Agaricomycotina	457.58 ± 0.69	352.97–563.05
C: Tremellomycetes/Agaricomycotina	406.17 ± 0.71	304.71–502.81
D: Dacrymycetes/Agaricomycetes	388.28 ± 0.70	294.39–483.74
E: Auriculariales/Agaricomycetes	316.85 ± 0.72	235.88–400.29
F: Trechisporales/Agaricomycetes	299.97 ± 0.71	220.04–376.82
G: Hymenochaetales/Agaricomycetes	283.21 ± 0.70	208.32–357.41
H: *Sanghuangporus*	30.85 ± 0.19	15.62–53.65
^∗^C1: Hymenochaetales	207.21 ± 0.56	146.74–265.46
^∗^C2: *Mycena*/*Marasmius*	123.57 ± 0.45	80.52–170.72

*Fossil record (for comparison): ^∗^C1, Quatsinoporites cranhamii, 125–130 Mya; ^∗^C2, Archaeomarasmius leggetti, 90 Mya (minimum age).*

**FIGURE 4:**
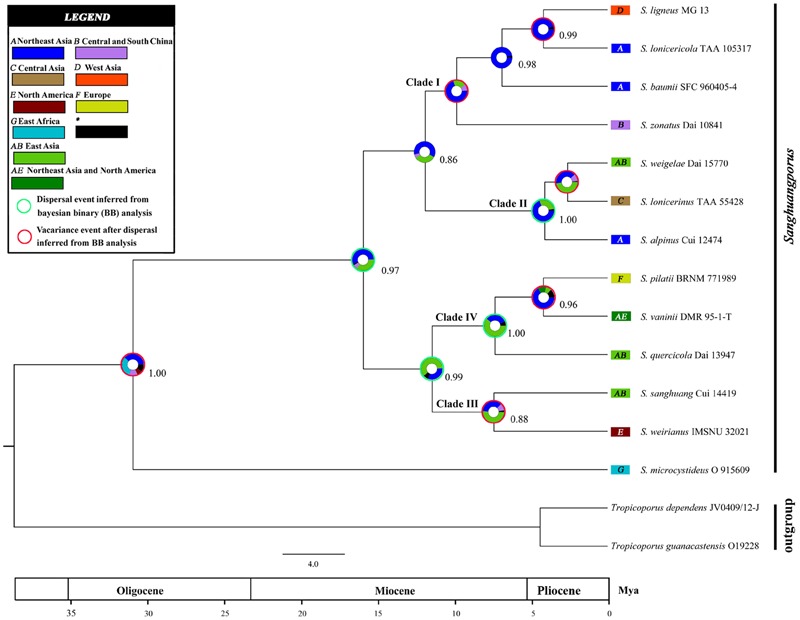
Divergence time estimation and ancestral area reconstruction of *Sanghuangporus* using the ITS dataset. The chronogram was obtained by molecular clock analysis using BEAST. The pie chart in each node indicates the possible ancestral distributions inferred from Bayesian Binary MCMC analysis (BBM) implemented in RASP. Bayesian credibility values (PP) over 0.85 are indicated near the pie chart of the tree. Green circle around pie charts indicate possible dispersal events, red circle indicate dispersal and vicariance events as suggested by BBM analysis.

**FIGURE 5:**
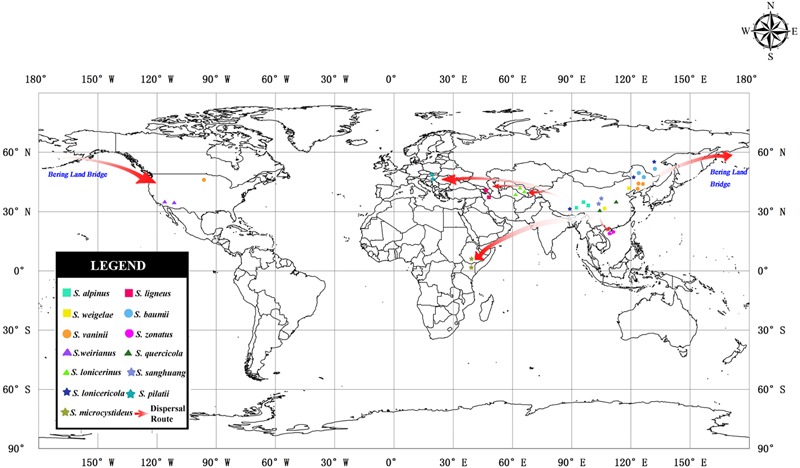
Map of the geographic distribution of *Sanghuangporus* and possible dispersal routes generated by ArcGIS v10.1. A hypothetical schematic depiction of the original locations, the migration routes the speciation of *Sanghuangporus*.

The inferred historical biogeographic scenarios from the analyses using RASP are shown in Figure 4 and summarized in Table 5. The Bayesian binary Markov chain Monte Carlo analysis shows that East Asia has the highest probability (38%) of being the ancestral area of *Sanghuangporus.* Northeast Asia was the most likely ancestral area for Group 1 ∖^∗^ ROMAN and Group 2 ∖^∗^ ROMAN 4 ∖^∗^ ROMAN, at 70 and 69%. The most likely ancestral area for Group 3 ∖^∗^ ROMAN (51%) and Group 4 ∖^∗^ ROMAN (61%) was East Asia. Ten dispersal events and six vicariance events are needed to explain the current distribution of the genus. The basal species (*S. microcystideus*) exhibited a tropical distribution pattern (Zhou and Wang, 2015). We estimated the crown node of *Sanghuangporus* to correspond to 30.85 ± 0.19 Mya. The Northeast Asian–East Africa lineages split during the end of the Mid-Miocene, which is consistent with the previous biogeographic studies of Tertiary relics (Zhou et al., 2012; Yang et al., 2016). Additionally, six kinds of dispersal routes were inferred: Northeast Asia–East Africa, Northeast Asia–Southern China, Northeast Asia–Western Asia, Northeast Asia–Europe, East Asia–North America, and East Asia–Central Asia (Figure 5).

**Table 5:** Estimated divergence times of the main groups correspond with the dating analysis of ITS datasets.

Node	Mean ± standard error	95% HPD
Clade I crown node	10.62 ± 0.02	6.22–15.60
Clade IV crown node	7.51 ± 0.02	3.70–11.44
Clade III crown node	7.40 ± 0.02	3.23–12.32
Clade II crown node	4.69 ± 0.01	1.71–8.33
*S. vaninii*/*S. pilatii*	4.46 ± 0.01	1.52–7.82
*S. weigelae*/*S. lonicerinus*	2.58 ± 0.01	0.90–5.64
*S. ligneous*/*S. lonicericola*	4.31 ± 0.01	1.63–7.35

## Discussion

The genus *Sanghuangporus* is one of the most important medicinal fungal genera. Currently, 13 species are accepted in *Sanghuangporus*. These species form four major clades (Figure 2). This study also shows that *Tropicoporus* is as a sister genus to *Sanghuangporus* (MP = 100%, ML = 100%, and BPP = 1.00).

Group A contains five *Sanghuangporus* species from Europe, East Asia, and North America. In the combined dataset topology (Figure 2), *S. vaninii, S. pilatii*, and *S. quercicola* are grouped together, with significant supports from the MP/ML/BI analyses (MP = 99%, ML = 98%, BPP = 1.00). These three species share several morphological synapomorphies including (1) a dark brown pileal surface and a buff-yellow pore surface; (2) similar dimitic hyphal structure; (3) basidiospores ovoid to broadly ellipsoid (Wu et al., 2012; Tomsovsky, 2015; Zhu et al., 2017). Nevertheless, *S. quercicola* grows on *Quercus*, but *S. pilatii* and *S. vaninii* mostly grow on *Populus* (Wu et al., 2012; Tomsovsky, 2015; Zhu et al., 2017). Therefore, these species have close relationships to each other except for differences in their host plants, suggesting a gene exchange in this clade. In addition, *S. weirianus* is restricted to living walnut substrata in Arizona, New Mexico, and Mexico (Gilbertson, 1979). *Sanghuangporus sanghuang* has brownish yellow basidiospores, and this species grows exclusively on *Morus* in East Asia (Wu et al., 2012). Moreover, the latter differs from the former in its shallower yellow basidiospores.

In Group B, *Sanghuangporus lonicericola, S. baumii, S. zonatus*, and *S. ligneous* form a weakly supported clade in our phylogenic analysis (MP = 67%, ML = 78%, BPP = 0.92) in Figure 2. To date, *S. zonatus* was only recorded from the tropics (Tian et al., 2013). It is characterized by an applanate pileus with an acute margin (Tian et al., 2013). The molecular data suggest a closer relationship between the East Asian species *S. lonicericola* and the West Asian species *S. ligneous*. Both have similar basidiomes and grow on *Lonicera*. This similarity may result from morphological stasis caused by similar refuges, hosts and habitats in the Quaternary Ice Age (Berbee and Taylor, 1992; Percudani et al., 1999; Djamali et al., 2012). In the East Asian species, *S. baumii* is often mistaken for *S. lonicericola*, but, *S. baumii* has slightly larger basidiospores (3.5–4.5 × 3.2–3.5 μm vs. 3.3–4.1 × 2.4–3.3 μm for *S. lonicericola*) and larger pores (5–7 per mm vs. 8–10 per mm for *S. lonicericola*) and grows mostly on *Syringa* (Wu et al., 2012).

Group C contains two East Asian species, *Sanghuangporus alpinus* and *S. weigelae*, and a Central Asia species, *S. lonicerinus*, and this group is well supported by the ML and BI analyses (ML = 100%, BPP = 1.00) and moderately supported by the MP analysis (MP = 77%). The two East Asian species form a strongly supported clade in the BI analysis (BPP = 0.99), but this was not supported in the ML analysis. *S. alpinus* is distinguished by its homogeneous context, but *S. weigelae* shows duplex context (Wu et al., 2012). In addition, the former species is presently only known from the plateau region, but the latter has a warm temperate to subtropical distribution (Tian et al., 2013). *S. lonicerinus* covers most parts of Central Asia and grows mostly on *Lonicera* (Wu et al., 2012). These species are distinct from those of the other clades in the phylogenetic tree (Figure 2).

Group D consists of only *Sanghuangporus microcystideus*. It resembles *S. alpinus* in having a dark brown pileal surface, but the basidiospores of *S. microcystideus* are slightly larger (5.1–6.0 × 4.1–5.0 μm). This East African species was collected on *Olea* (Zhou et al., 2016). In the phylogenetic analysis (Figure 2), Group D formed a significantly supported terminal lineage (MP = 100%, ML = 98%, BPP = 1.00).

The maximum crown age of *Sanghuangporus* was estimated to be around the Oligocene (30.85 ± 0.19 Mya) (Figure 3, 4), and Northeast Asia was inferred as the most likely (38%) ancestral area (Figure 4). The changes caused by tectonic activity and dramatic climate change since the collision between the Arabian plate and the Eurasian closed the Tethys Sea. Asia and Africa were connected via the “Gomphotherium Landbridge” during the late Oligocene (Rögl, 1999, 2001). Expanded tropical forests associated with the Miocene warming trend may be an important factor for species exchanges between Asia and Africa (Yuan et al., 2005). It is probable that the common ancestor between Asia and Africa migrated via the expanded rainforests at the end of the Mid-Miocene. Later, global cooling and increasing aridification (16–2.8 Mya) replaced the Mid-Miocene climate optimum (approximately 15 Mya) after the temperature began to fluctuate in the Miocene–Oligocene boundary (approximately 24 Mya) (Zachos et al., 2001; Morley, 2003; Guo et al., 2008; Besnard et al., 2009; Chen et al., 2014). As a consequence, the expansion of the grassland vegetation gradually replaced tropical forests during the Quaternary ice ages. Climatic shifts would exacerbate the depth of divergence between sister lineages (Godfrey, 2000; Fleagle and Gilbert, 2006). This biogeographic distribution pattern was also identified in *Searsia, Lychnis, Uvaria*, and it is associated with the “out-of-Africa” dispersal of primates (Fleagle and Gilbert, 2006; Popp et al., 2008; Zhou et al., 2012; Yang et al., 2016). With repeated climatic oscillations and the major growth of the Arctic Ice Sheet, there was climate deterioration in the late Pliocene and Pleistocene in Europe, Asia, and North America (Godfrey, 2000; Shawn and John, 2006). These severe climatic oscillations affected environmental heterogeneity and produced great changes in *Sanghuangporus* species distributions in the Northern Hemisphere.

According to the molecular clock results, the separation of Clade I and Clade II ∖^∗^ ROMAN initially occurred at 12.47 Mya (Figure 4). The estimated divergence time coincides with the climate changes in the middle Miocene, probably following the uplift of the Himalaya-Tibetan Plateau (Zhang and Fritsch, 2010). As a consequence, increasingly East Asian monsoons served as a key trigger of the aridification of central China (Molnar et al., 2010; Li et al., 2011; Zhao et al., 2012; Chatterjee et al., 2013; Zhang et al., 2015). Two sister clades within this lineage exhibited independent east-to-west dispersal in the ancestral area reconstruction analyses (Figure 4, 5). In Clade I, the divergence between the West Asia species *Sanghuangporus ligneous* and its East Asian counterpart (*S. lonicericola*) is estimated to have occurred approximately 4.31 Mya in the Pliocene, and East Asia is inferred to be the common ancestral area. A complex sequence of tectonic setting deformation occurred, leading to the uplift of the mountain ranges starting in the Late Miocene (Axen et al., 2001; Eronen et al., 2010; Djamali et al., 2012). The variation event might indicate a relatively stable climate during the late Miocene-Pliocene compared to the Quaternary period that caused small species range shifts and gene flow in the Iranian plateau (Sherkatia and Letouzeyb, 2004; Ritz et al., 2006). In Clade 2 ∖^∗^ ROMAN, three *Sanghuangporus* species from East Asia and Central Asia are closely related (Figure 2, 4). We inferred vicariance events within Central Asia during the Pliocene and early Pleistocene (2.58 Mya). Palaeogeographical evidence suggests a dramatic change from a region with a humid climate to a semi-arid climate in the western part of central Asia (Manafzadeh et al., 2014; Wang et al., 2016; Lauer et al., 2017). This change is thought to have spurred speciation as a result of habitat shifts promoted by progressive aridification and the Quaternary glacial-interglacial cycles, which started approximately 2.6 Mya at the beginning of the Quaternary (Gibbard et al., 2009; Zhang and Zhang, 2012). Hence, the climatic shifts caused range contraction and speciation in the region.

Our results indicate that two independent intercontinental distribution patterns (Clade 3 ∖^∗^ ROMAN and Clade 4 ∖^∗^ ROMAN) started to diversify during the Miocene (Figure 4). Clade 3 ∖^∗^ ROMAN is divided into two sister species, *Sanghuangporus sanghuang* and *S. weirianus* (Figure 4), and this clade had an intercontinental distribution between East Asian and North America. The divergence of Clade III and Clade IV occurred before the Bering Land Bridge (BLB) was detached approximately 5.5 Mya (Milne, 2006). We speculate that intercontinental transfers of the *Sanghuangporus* ancestor occurred between East Asia and North America via the BLB. Estimated vicariance events between North America and Asia in the late Miocene (7.51 Mya) are also consistent with biogeographic patterns supported by the bidirectional floristic exchange in the temperate taxa (Manos and Stanford, 2001). The vicariance due to reduced precipitation may be responsible for the endemism of *Sanghuangporus* in America. Dispersal-vicariance theory is used to understand the intercontinental migration patterns (Donoghue and Smith, 2004; Cai et al., 2014). In addition, in Clade 4 ∖^∗^ ROMAN, the estimated divergence time of *S. vaninii* is consistent with the known dispersal of the host plant (*Populus*) from Asia to North America (Godfrey, 2000; Du et al., 2015). Palaeobotanical data indicate that the BLB route played a crucial role in plant dispersal for *Populus, Lonicera, Leibnitzia*, and *Linnaea* (Baird et al., 2010; Smith and Donoghue, 2010; Du et al., 2015; Wang et al., 2015). The notable finding is that the biogeographical pattern of the European species (*S. pilatii*) and the Northeast Asian and North American species (*S. pilatii*) nested in the East Asia lineage, which would be expected given the probable direction of ancestor migration. The estimated crown age is approximately 4.46 Mya between *S. pilatii* and *S. vaninii*. Pliocene climatic events caused range contraction, fragmentation, and the extinction of numerous species, which accelerated the intra-phylogroup diversification processes in *Sanghuangporus*. Long-distance dispersal has also been detected in other fungi, such as *Bondarzewia* Singer (Bondarzewiaceae, Russulales) (Song et al., 2016). Our data suggest that the other East Asian species in this clade, *S. quercicola*, diversified approximately 7.44 Mya. Afterward, the ice sheets expanded in the Northern Hemisphere (Chang et al., 2017). Divergence due to habitat fragmentation in the late Miocene is thought to be responsible for the endemism of *S. quercicola*. The divergence events exhibited by the plant taxa (*Quercus glauca* and *Pinus armandii*) confirm the geological events associated with species diversification in subtropical Asia (Andrew and Hall, 2006; Xu et al., 2015).

We found that the samples of *Sanghuangporus* are scant in some parts of the world, such as South America, the Indian Subcontinent and Australia. Therefore, the further sampling is required to clarify the population history of these areas.

## Author Contributions

B-KC and LZ designed the experiments. LZ, JSi, and BK-C prepared the samples. LZ, JSo, and JL-Z conducted the molecular experiments and analyzed the data. LZ, JSi, JL-Z, JSo, and BK-C drafted the manuscript. All of the authors approved the manuscript.

## Conflict of Interest Statement

The authors declare that the research was conducted in the absence of any commercial or financial relationships that could be construed as a potential conflict of interest.

## Footnotes

1 http://www.ncbi.nlm.nih.gov/

2 www.treebase.org

3 http://tree.bio.ed.ac.uk/software/tracer/

4 http://esri.com/arcgis

## Abbreviations

BBM: Bayesian binary Markov chain Monte Carlo
BI: Bayesian inference
BJFC: Herbarium of the Institute of Microbiology, Beijing Forestry University
BPP: Bayesian posterior probabilities
CI: consistency index
EF1-α: translation elongation factor 1-α
GTR-I+G: general time reversible+proportion invariant+gamma
HI: homoplasy index
HPD: higher posterior densities
IFP: Herbarium of the Institute of Institute of Applied Ecology, Chinese Academy of Sciences
ITS: internal transcribed spacer
ML: maximum likelihood
MP: maximum parsimony
Mya: million years ago
nrLSU: nuclear large subunit rDNA
RC: rescaled consistency index
RI: retention index
RPB1: DNA-directed RNA polymerase II subunit 1
RPB2: DNA-directed RNA polymerase II subunit 2
TL: tree length
